# Enhanced chemoselectivity of a plant cytochrome P450 through protein engineering of surface and catalytic residues

**DOI:** 10.1007/s42994-021-00056-z

**Published:** 2021-08-10

**Authors:** Xiaopeng Zhang, Wei Luo, Yinying Yao, Xuming Luo, Chao Han, Yang Zhong, Bo Zhang, Dawei Li, Lida Han, Sanwen Huang, Per Greisen, Yi Shang

**Affiliations:** 1 Shenzhen Branch, Guangdong Laboratory of Lingnan Modern Agriculture, Genome Analysis Laboratory of the Ministry of Agriculture and Rural Affairs, Agricultural Genomics Institute at Shenzhen, Chinese Academy of Agricultural Sciences, Shenzhen, 518116 China; 2 Yunnan Key Laboratory of Potato Biology, The CAAS-YNNU-YINMORE Joint Academy of Potato Sciences, Yunnan Normal University, Kunming, 650500 China; 3 National Key Laboratory of Crop Genetic Improvement and National Centre of Plant Gene Research, Huazhong Agricultural University, Wuhan, 430070 China; 4 Biotechnology Research Institute, Chinese Academy of Agricultural Sciences, Beijing, 100081 China; 5 Key Laboratory of Biology and Genetic Improvement of Horticultural Crops of Ministry of Agriculture, Sino-Dutch Joint Lab of Horticultural Genomics, Institute of Vegetables and Flowers, Chinese Academy of Agricultural Sciences, Beijing, 100081 China; 6 Novo Nordisk Research Center Seattle Inc, Seattle, WA 98109 USA

**Keywords:** Plant cytochrome P450, Protein engineering, Rosetta, Amino acid co-evolution, Surface residue

## Abstract

Cytochrome P450s (P450s) are the most versatile catalysts utilized by plants to produce structurally and functionally diverse metabolites. Given the high degree of gene redundancy and challenge to functionally characterize plant P450s, protein engineering is used as a complementary strategy to study the mechanisms of P450-mediated reactions, or to alter their functions. We previously proposed an approach of engineering plant P450s based on combining high-accuracy homology models generated by Rosetta combined with data-driven design using evolutionary information of these enzymes. With this strategy, we repurposed a multi-functional P450 (CYP87D20) into a monooxygenase after redesigning its active site. Since most plant P450s are membrane-anchored proteins that are adapted to the micro-environments of plant cells, expressing them in heterologous hosts usually results in problems of expression or activity. Here, we applied computational design to tackle these issues by simultaneous optimization of the protein surface and active site. After screening 17 variants, effective substitutions of surface residues were observed to improve both expression and activity of CYP87D20. In addition, the identified substitutions were additive and by combining them a highly efficient C11 hydroxylase of cucurbitadienol was created to participate in the mogrol biosynthesis. This study shows the importance of considering the interplay between surface and active site residues for P450 engineering. Our integrated strategy also opens an avenue to create more tailoring enzymes with desired functions for the metabolic engineering of high-valued compounds like mogrol, the precursor of natural sweetener mogrosides.

**Supplementary Information:**

The online version contains supplementary material available at 10.1007/s42994-021-00056-z.

## Introduction

By constantly adapting to changing environments, plants can produce a vast array of specialized metabolites such as alkaloids, terpenoids, and phenols, to mediate environmental interactions. These structurally and functionally diverse compounds also constitute valuable sources for the development of pigments, cosmetics, pesticides, and pharmaceuticals (Kroymann 2011; Wink 2018). In the biosynthesis of these chemicals, their carbon skeletons are mainly produced by signature enzymes such as cyclases, and further modified by tailoring enzymes such as cytochrome P450s (P450s), acyltransferases, and glycosyltransferases, to produce the final products (Nützmann and Osbourn 2014). Among these tailoring enzymes, P450s are the most versatile catalysts, and are widely utilized to catalyze various reactions including: regio- and stereoselective oxidations, deamination, decarboxylation, C–C cleavage, ring expansion, and dehydration that are considered challenging or even impossible with traditional chemical synthesis (Jung et al. 2011; Guengerich and Munro 2013). Given their irreplaceable roles, P450s are of great importance for the metabolic engineering of plant metabolites in heterologous hosts such as bacteria or yeast (Urlacher and Girhard 2019).

Currently, multi-omics tools have been widely applied in the search for enzymes or regulatory factors involved in plant metabolic pathways (Fernie and Gutierrez-Marcos 2019; Shang and Huang 2018). Unfortunately, P450s form ~ 1% of coding genes in plant genomes (Nelson and Werck-Reichhart 2011), making it challenging to screen for target genes from genome-wide sequencing due to their high redundancy. Additionally, the heme containing P450s are commonly coupled with redox partners, e.g., cytochrome P450 reductase (CPR), to catalyze oxidation reactions, further complicating the functional characterization of P450s. In this situation, protein engineering strategies including directed evolution (DE) and rational design are applied as complementary tools in the functional studies of plant P450s, and to create P450s with a specific functionality, alongside exploring the mechanisms of P450-mediated reactions (Mcintosh et al. 2014; Renault et al. 2014; Urlacher and Girhard 2019). Multiple non-natural reactions have been created through DE of P450s (Arnold 2015). However, as high-throughput screening methods are required for DE of enzymes (Bassalo et al. 2016; Jung et al. 2011), this strategy can in practice be difficult for complicated chemical reactions. In addition, P450s, especially those from eukaryotes, are usually membrane-anchored proteins that are exceedingly hard to crystallize (Jung et al. 2011). Although modeling-based mutagenesis of CYP enzymes could be applied to alter their products (Ignea et al. 2015; Scheler et al. 2016), it remains challenging to efficiently engineer plant P450s through rational design.

**Fig. 1:**
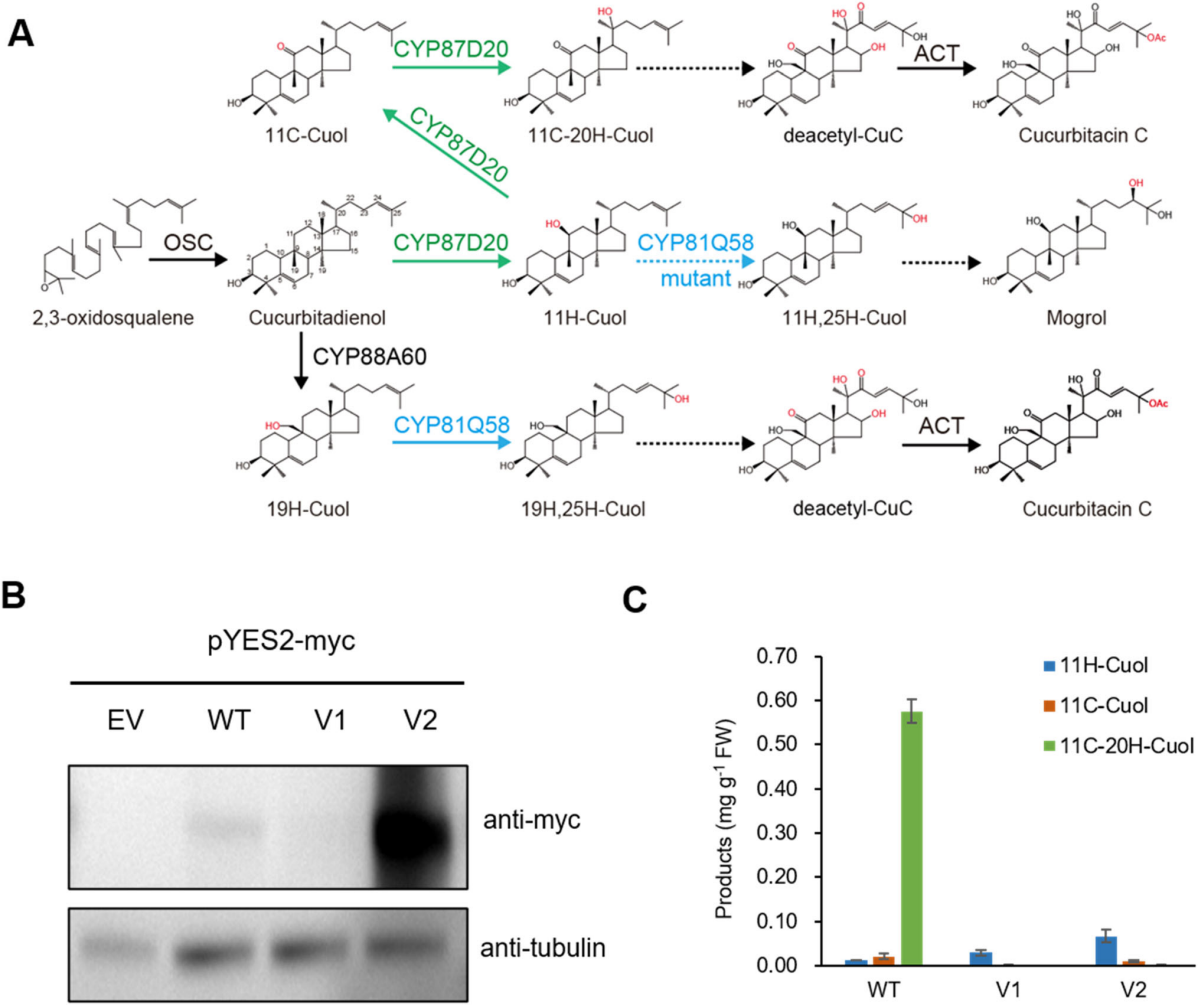
Increased expression of the CYP87D20 variant V2 with a single substitution. **A** Two plant P450s (CYP87D20 and CYP81Q58) are involved in the cucurbitacin C (CuC) biosynthetic pathway, where modification of these catalysts could be used to create a de novo pathway to produce mogrol. *H* hydroxyl, *C* carbonyl, *Cuol* cucurbitadienol, *OSC* oxidosqualene cyclase, *ACT* acyltransferase. **B** Protein expression levels of CYP87D20 and its variants in yeast. The target protein and its internal control (tubulin) expressed in the yeast harboring empty vector (EV), CYP87D20 (WT), variant 1 (V1, L48F-S49A-I61F-L120T-T352K-L356P), or variant 2 (V2, V1-K352I) were detected using Western blotting. A single substitution K352I improved the expression of the target protein in yeast. **C** Comparisons of the metabolite yield in the yeast expressing CYP87D20 or its variants. 11H-Cuol was the target product, while 11C-Cuol and 11C-20H-Cuol were two by-products. Contents of these compounds were quantified using a standard curve (see method). Data are presented as means ± SD (*n* = 3 biological repeats)

Previously, we built a data-driven iterative pipeline for engineering plant enzymes such as oxidosqualene cyclase and P450 (Li et al. 2019; Ma et al. 2016). Briefly, this method combines structural design with the phylogenetic analysis of both conserved and co-evolved enzyme residues. With advances in DNA sequencing technology and the dramatically decreased cost, there is an abundance of plant genomic data now available (Cheng et al. 2018), that can aid in capturing both conserved and co-evolving amino acids in protein families (Kamisetty et al. 2013). Combining the phylogenetic analysis with computational protein design, reduces the number of variants to be experimentally tested (Huang et al. 2016; Khare et al. 2012). To test the efficiency of this data-driven iterative approach in engineering plant P450s, two P450s (CYP87D20 and CYP81Q58) involved in the C11 and C25 oxidations of the cucurbitane backbone in cucurbitacin C (CuC, the major bitter compound in cucumber) pathway (Shang et al. 2014; Zhou et al. 2016), were selected as targets. Given that both C11 and C25 hydroxylations of the same backbone are also required to produce mogrol, the precursor of natural non-sugar sweetener mogrosides identified from the mature fruit of *luo-han-guo* (*Siraitia grosvenorii*, monk fruit) (Itkin et al. 2016), we aim to create a de novo pathway to re-direct the metabolic flux from the CuC biosynthesis toward mogrol by engineering the target enzymes (Fig. 1A). In a proof-of-concept study (Li et al. 2019), the multi-functional P450 (CPY87D20) was successfully converted into a monooxygenase, and participated in the novel pathway to produce mogrol. However, during the engineering process, we noticed that one of the variants, V1 (L48F-S49A-I61F-L120T-T352K-L356P), had a very low expression level but a relatively high catalytic efficiency in yeast (Fig. 1B and C). This suggested that improving the expression level of this variant could further increase its catalytic capacity.

In this study, after screening 17 variants, beneficial substitutions of both surface and active residues were identified which improved both protein expression as well as catalytic activity in yeast. Furthermore, when these mutations were combined, we were able to create a highly efficient C11 hydroxylase of cucurbitadienol. This study shows the importance of distal substitutions to compensate for active site modifications in plant P450s engineering.

## Results

### A single residue substitution improves expression of CYP87D20 variant in yeast

While investigating the computational model of V1, it was observed that the T352K mutation introduced a positive charge into the hydrophobic active pocket, which could decrease protein stability (Fig. S1A). In addition, the Position Specific Scoring Matrix (PSSM) revealed that beta-branched amino acids, like valine (V) or isoleucine (I), were more favored than lysine (K) at this position in the CYP87 family (Fig. S1B). Since isoleucine is bulkier than valine and threonine, we introduced the single substitution K352I into V1, to generate a new variant, V1-K352I (V2). After co-expressing V2 with a CPR redox partner (*Csa1G423150*) in yeast cells for 48 h, the yeast metabolic extract was analyzed with high-performance liquid chromatography (HPLC) coupled with high-resolution quadrupole time-of-flight mass spectrometry (qTOF) at atmospheric pressure chemical ionization (APCI). As shown in Fig. 1, this substitution dramatically improved the protein expression and the production of target compound, 11-hydroxyl cucurbitadienol (11H-Cuol), in yeast cells (Fig. 1B and C).

### Activity increase of C11 hydroxylase of cucurbitadienol by changing surface residues

**Fig. 2:**
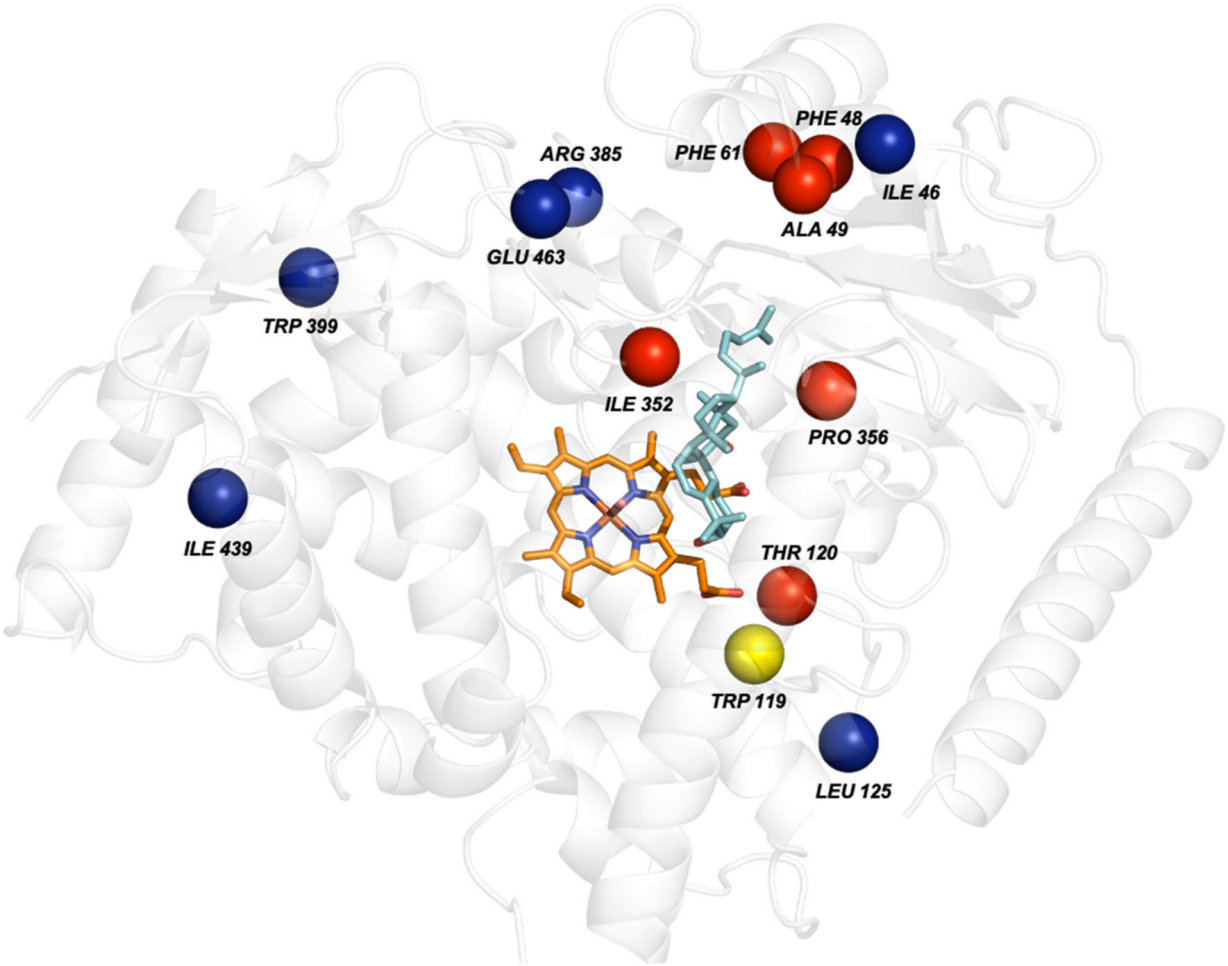
Computational model with substrate, cofactor and selected sites for V2 engineering. The red spheres show the sites mutated to generate V2 (L48F-S49A-I61F-L120T-K352I-L356P) with substrate and cofactor docked into the active site. The surface and active positions selected for V2 engineering are shown by blue and yellow spheres, respectively. The P450 is shown in cartoon, 11H-Cuol in cyan sticks; the heme group with a coordinating oxygen is shown in sticks with carbons copper colored, nitrogen blue, oxygen red, and iron orange

To further improve the efficiency of V2, we next expanded our study to protein surface engineering using the method described previously (Li et al. 2019). PSSM and Gremlin were used to identify the conserved and co-evolved positions within V2, respectively. Among the 41 amino acid substitutions with positive PSSM values, 27 of them are located on the protein surface, while the remaining are located in or around the active pocket (Table S1; Figure S2A). Of the 41 substitutions of the co-evolved amino acids, 28 were located on the protein surface (Table S2; Fig. S2B). Overall, the 82 variants were clustered into eight groups based on phylogenetic analysis, and the lowest-scored variant of each cluster was chosen for experimentally testing (Fig. S3). Six surface residues (I46L, L125D, R385Y, W399K, I439H, and E463P), one semi-exposed residue (A49L), and one active site (W119I) were selected for experimental investigation (Fig. 2). Four of these substitutions were close to one another in the sequence and were merged to generate double-mutated variants: V2-I46L-A49L, and V2-W119I-L125D.

**Fig. 3:**
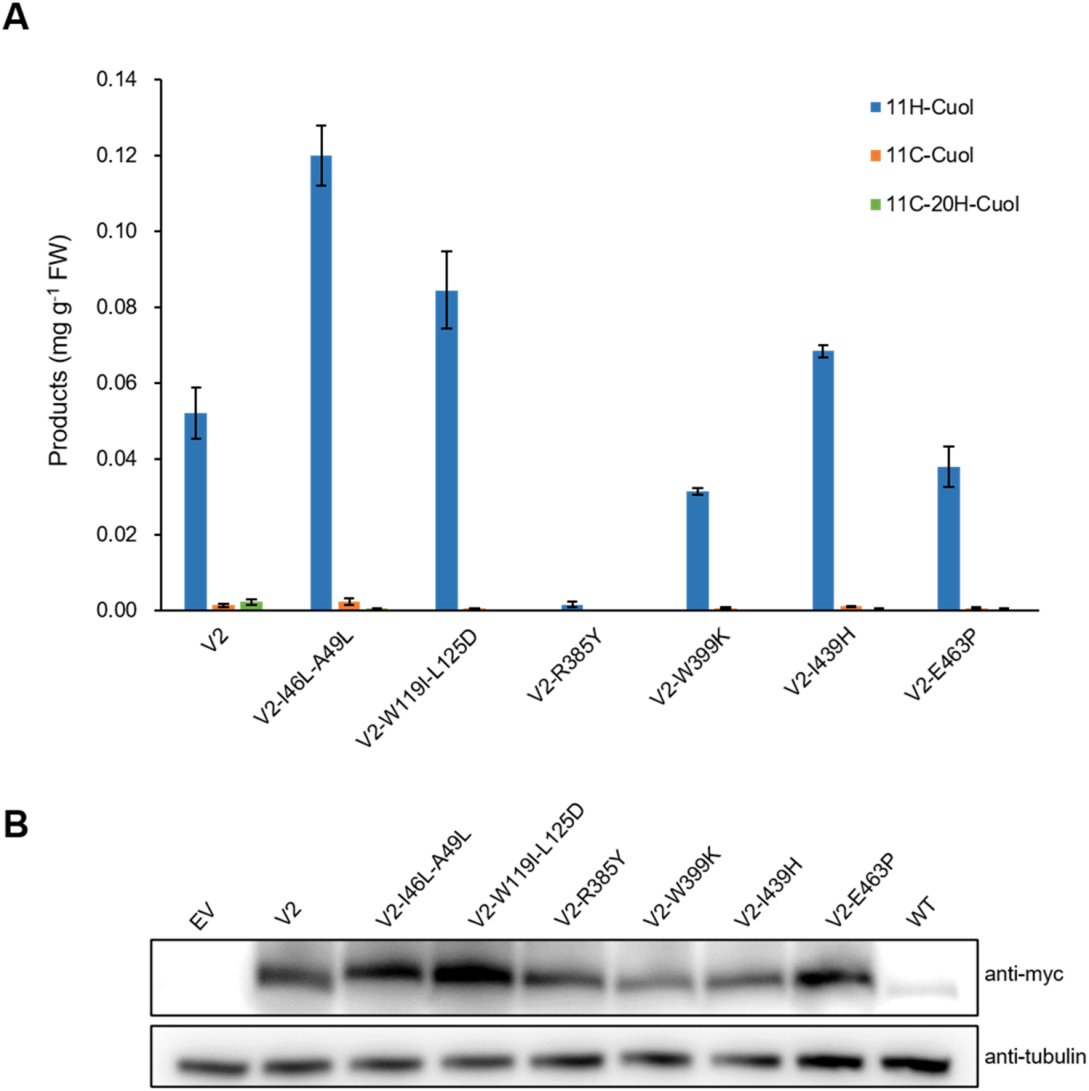
Increased yield of 11H-Cuol after optimization of V2. **A** The double mutant, V2-I46L-A49L, doubled the production of 11H-Cuol compared to V2. Two other variants, V2-W119I-L125D and V2-I439H, have improved yields compared with V2. The substitution of surface residue, R385Y, abolished enzyme activity. Data are presented as means ± SD (*n* = 3 biological repeats). **B** Protein expression levels of CYP87D20 and its six variants in yeast by Western blotting

Interestingly, the variant V2-I46L-A49L shows similar protein expression level to V2, but produced twice as much 11H-Cuol than V2 in yeast (Fig. 3). According to the computational model, the A49L substitution is located at the entrance to the active center of P450, and may be involved in substrate packing. Thus, this mutation could stabilize the substrate orientation to enhance chemoselectivity of the variant (Fig. S4). The I46L substitution is located in a helical area, and altering the beta-branched isoleucine to a non-branched leucine could stabilize this helical region of the enzyme (Fig. S4). Another substitution at the surface, R385Y, abolished the catalytic ability of V2-R385Y, although its expression level remained comparable to V2 (Figs. 2; 3). These results confirmed that mutating surface residues on plant P450 can significantly influence its catalytic activity without affecting its expression. The remaining variants (V2-W119I-L125D, V2-W399K, V2-I439H, and V2-E463P) maintained comparable protein levels, as well as catalytic activity to V2 (Fig. 3), indicating that these positions are not key elements for repurposing CYP87D20.

**Fig. 4:**
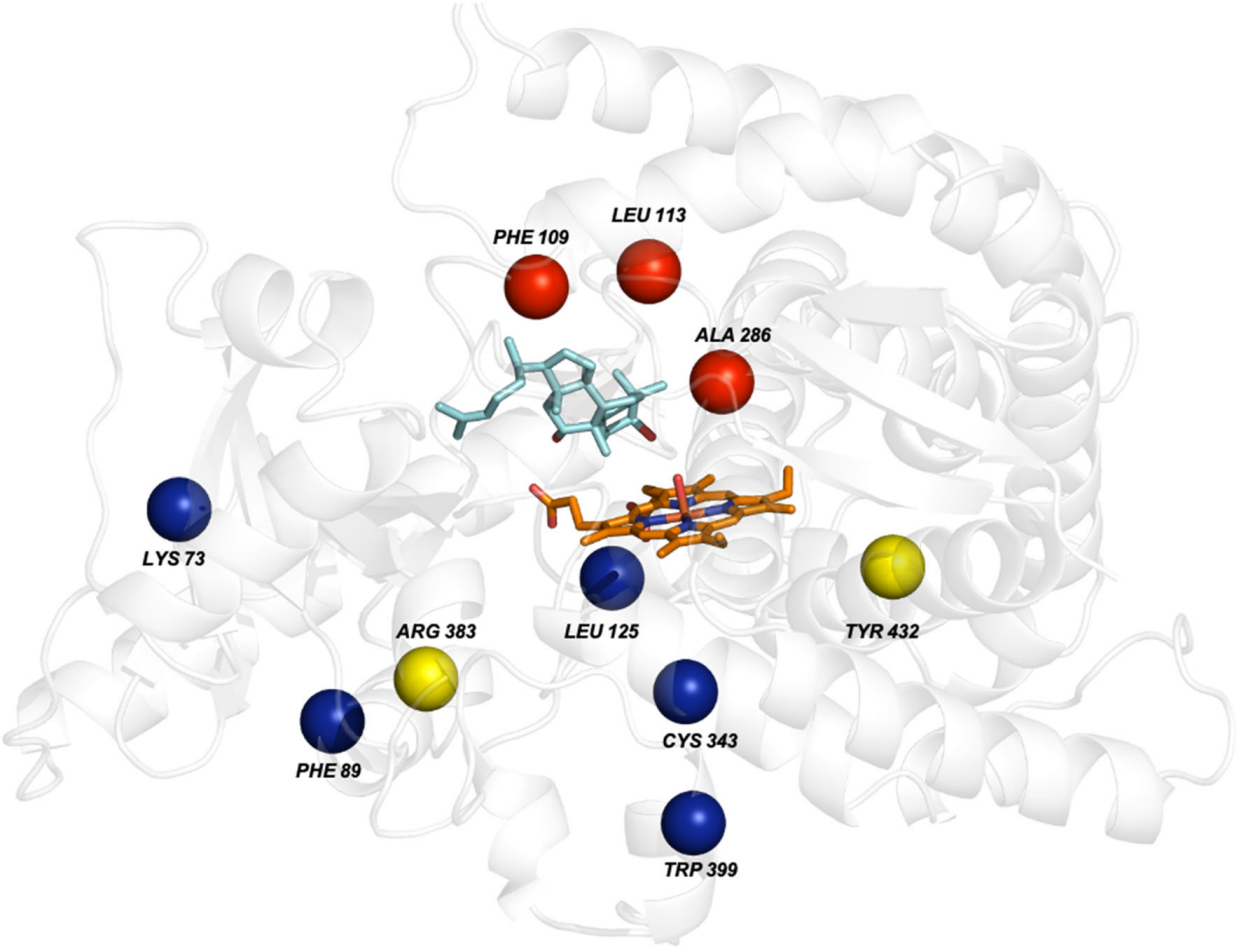
Sites selected for V3 engineering. The red spheres represent the original mutation sites of V3 (L109F-F113L-E286A). The five blue spheres and two yellow spheres, respectively, represent surface and interior mutations. 11H-Cuol, cyan sticks; the heme group with a coordinating oxygen is shown in sticks with carbons copper colored, nitrogen blue, oxygen red, and iron orange

**Fig. 5:**
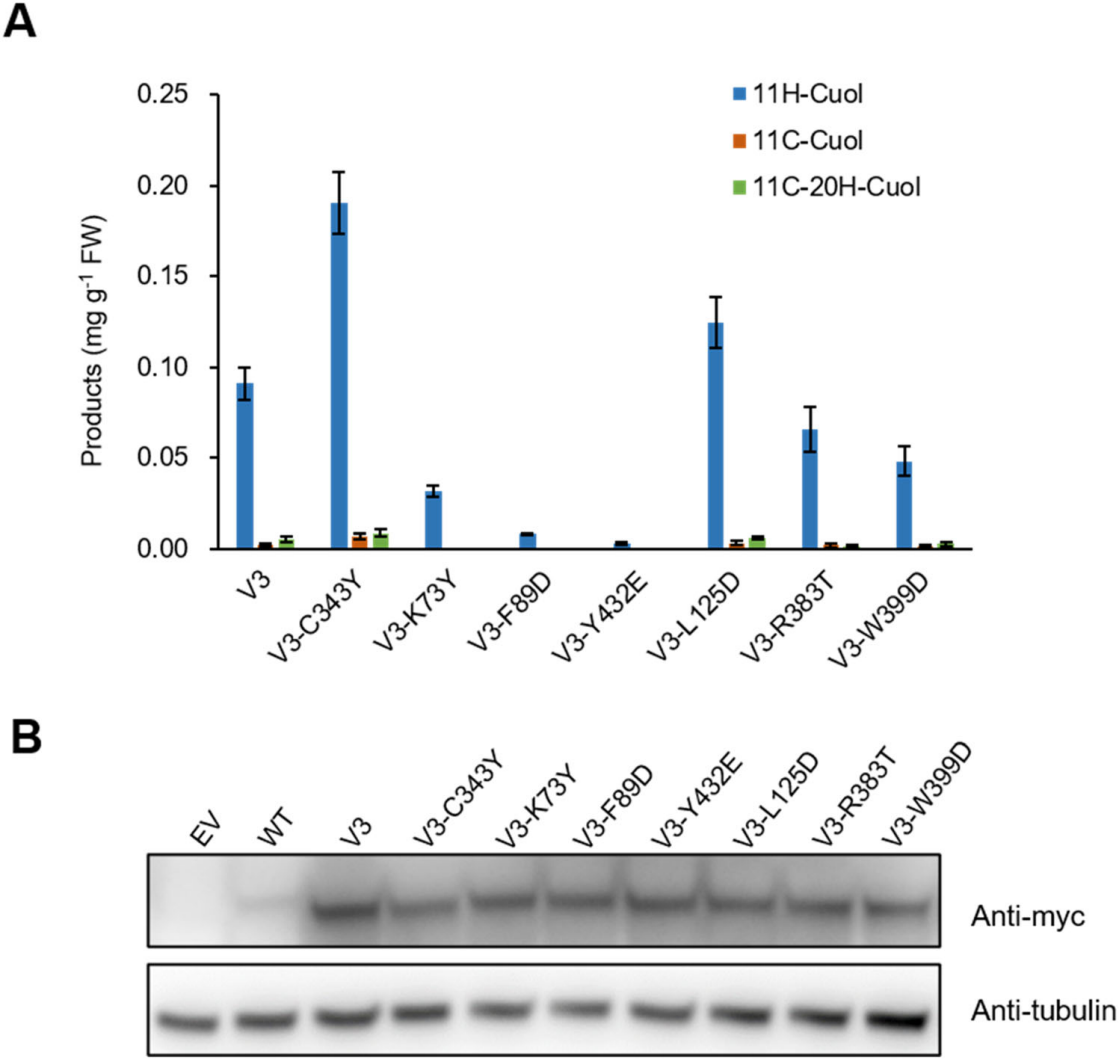
Increased yield of 11H-Cuol after optimization of V3. **A** The surface mutation C343Y increased the yield of 11H-Cuol produced compared to V3. Two surface mutations (K73Y and F89D), and one active center mutation (Y432E) severely abolished the catalytic ability. Data are presented as means ± SD (*n* = 3 biological repeats). **B** Protein expression levels of CYP87D20 and its seven variants in yeast by Western blotting. All the variants had lower, or similar expression levels compared with V3

In our previous study, another CYP87D20 variant V3 (L109F-F113L-E286A), exhibited a high ability to produce 11H-Cuol (Li et al. 2019), making V3 another suitable option for further engineering. Five of the seven substitutions from the evolutionary analysis were located on the protein surface, while the remaining two were in the pocket of the protein (Fig. 4; Table S3). The introduction of the surface mutation C343Y to V3 (V3-C343Y) further increased its capacity to produce 11H-Cuol, although its protein expression remained lower than V3 (Fig. 5; Fig. S5A). Substitutions of two other surface residues (K73Y, V3-K73Y; F89D, V3-F89D), and one interior site (Y432E, V3-Y432E), severely abolished catalytic activity (Fig. 5; Fig. S5B). Three other variants (V3-L125D, V3-R383T, and V3-W399D) were benign and revealed similar activity and expression levels as V3 (Fig. 5).

### Addition of substitutions creates a highly efficient C11 hydroxylase of cucurbitadienol

**Fig. 6:**
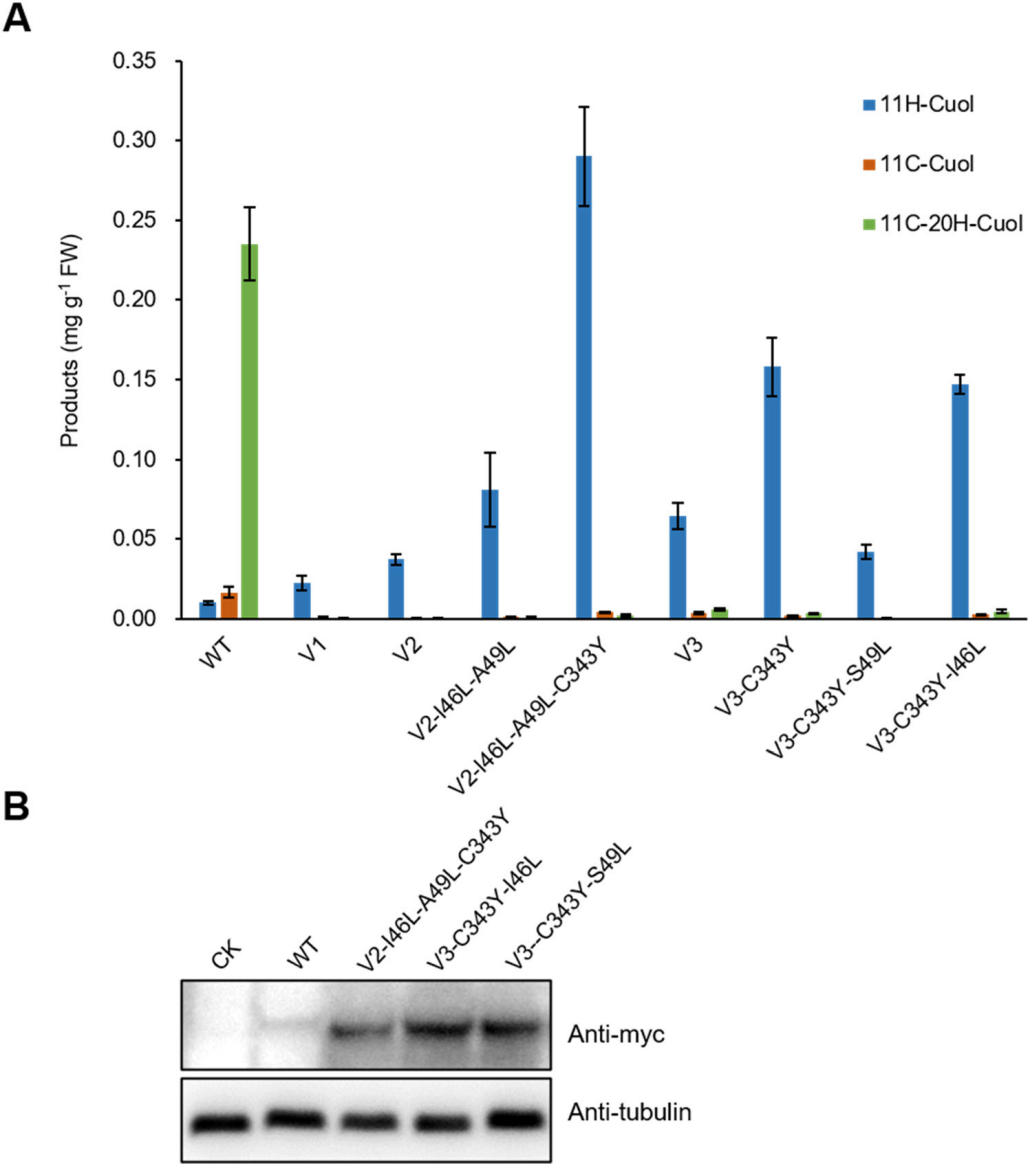
Generation of highly efficient C11 hydroxylase of cucurbitadienol by addition of beneficial mutations. **A** Products of the three target compounds produced in the yeast. Adding the two surface substitutions (C343Y and I46L) and a semi-exposed substitution (A49L) to V2 creates the most active C11 hydroxylas of cucurbitadienol. Data are presented as means ± SD (*n* = 3 biological repeats). **B** Protein expression levels of CYP87D20 and its variants in yeast. The protein expression levels of the variants are higher compared with WT

To further increase the yield of the desired product, 11H-Cuol, the beneficial substitutions were combined to improve the catalytic activity of these variants. The most promising variants, V2-I46L-A49L and V3-C343Y, produced ~ two fold higher C11 hydroxylation compared with their respective starting sequences. Thus, the beneficial substitution (C343Y) from V3 was introduced into V2-I46L-A49L to create a new variant, V2-I46L-A49L-C343Y. Interestingly, this variant created by adding two surface residue substitutions (C343Y and I46L) and a semi-exposed residue substitution (A49L) to V2, produced the highest yield of 11H-Cuol (approximately 3.6 and 28 fold higher than V2-I46L-A49L and WT, respectively) (Fig. 6; Table 1). In addition, this variant also had a much higher expression level than that of WT in yeast (Fig. 6). Similar procedure was performed for V3-C343Y, by adding the beneficial substitutions (I46L and A49L) identified from V2 engineering. However, these two novel variants, V3-C343Y-I46L and V3-C343Y-S49L, did not show any increased activity (Fig. 6). Given that V2 and V3 have different starting sequences, these results show the importance of introducing compensating or stabilizing mutations to incorporate additional beneficial mutations.

**Table 1:** Normalization of each product with respect to the total yield

Variants	11H-Cuol (%)	11C-Cuol (%)	11C-20H-Cuol (%)
WT	3.84	6.34	89.81
V1	95.22	3.83	0.96
V2	96.78	2.24	0.98
V2-I46L-A49L	97.81	1.63	0.57
V2-I46L-A49L-C343Y	97.85	1.46	0.70
V3	87.54	4.89	7.57
V3-C343Y	96.70	1.26	2.04
V3-C343Y-S49L	99.14	0.61	0.25
V3-C343Y-I46L	95.40	1.67	2.93

## Discussion

Since plant specialized metabolites constitute a valuable source for practical applications, there is a significant interest in large-scale production of these high-valued compounds. An often used strategy in the metabolic engineering of plant natural products, is to migrate metabolic pathways from plant to yeast or bacteria. However, as plant P450s are membrane-anchored proteins adapted to the micro-environments of plant cells, expression of these enzymes in heterologous hosts usually results in low expression or loss of activity. Strategies such as regulation of P450s expression, protein engineering, and co-expression of P450 and CPR, are usually applied to increase the yield of the target compound (Jiang et al. 2021; Shang and Huang 2020).

Our proposed strategy utilizes protein engineering as a method of optimizing plant P450s in yeast, either for its intended products or to create de novo pathways for novel metabolites. Using this computational protocol described earlier (Li et al. 2019; Ma et al. 2016), both issues can be addressed to adjust the enzyme to new micro-environment and/or to increase its catalytic activity. For some chemical reactions, it is not always possible to generate a high-throughput assay to test large numbers of variants. However, by applying computational algorithms (phylogenetic analysis and structure-based calculations in a data-driven fashion), it is possible to generate a limited number of variants for experimental testing and to iteratively improve desired properties. Smart or computational directed libraries are needed to limit the size of variants and have shown their value in other classes of proteins (Cherny et al. 2013). Here, by testing a limiting number of variants, we have achieved obvious improvements in enzyme expression, activity as well as specificity with our most promising variant.

Intriguingly, most of the identified improvements were surface residues or residues distal to the catalytic center. Since the protein expression levels of the variants with surface substitutions were comparable with V2 or V3, this suggests that mutating surface amino acids of plant P450 oxygenases could significantly influence their catalytic activity, and not only in increasing expression levels. Tuning of enzyme activity by altering surface residues has been observed by comparing cold and warm active trypsin (Isaksen et al. 2016). Since the differences in the micro-environments between plants and yeast are significant, surface residues should be included in the protein engineering process. In addition, our study also showed the importance of compensatory mutations (Aakre et al. 2015). This again stresses the importance of compensating the activity-promoting substitutions with the protein stabilizing ones to assure both expression and catalytic activity of plant enzymes are not compromised, especially when they are heterologously expressed under new physical–chemical conditions.

One thing needs to be noticed is that although all the variants have higher protein expression levels in yeast after engineering, their ability to produce 11H-Cuol are comparable or even less than WT to generate its native product 11C-20H-Cuol. The initial aim to create a monooxygenase has been achieved to increase flux in the de novo pathway toward mogrol, but its full potential is probably not yet realized. In addition, as yeast status in each assay was not exactly the same due to unexpected differences of yeast culturing, this minor difference would affect the measured catalytic activities of WT and its variants. To further understand the improvements of the different variants, more accurate in vitro methods should be adopted in future studies to get a deeper insight into the improved kinetic parameters.

In summary, our strategy works efficiently to engineer a plant P450 by consideration of both surface and active site residues. After testing 17 variants, a highly efficient C11 hydroxylase of cucurbitadienol was created. This enzyme can be used as the first tailoring enzyme in the de novo mogrol pathway.

## Materials and methods

### Generation of atomistic models of V2 and V3

The computational models of the variants V2 and V3 were created as previously described (Li et al. 2019; Song et al. 2013). To generate novel variants, we inserted the mutations and relaxed the model using RosettaScripts (Elizabeth et al. 2011; Fleishman 2011). The ligands (heme and substrate cucurbitadienol) were docked into the structure by Rosetta Ligand-Docking (Meiler and Baker 2006). All scripts are provided in the supplementary information.

### Phylogenetic analysis of variants

The amino acid conservation of V2 and V3 was computed using PSIBLAST 2.2.27 + to generate a Position Specific Scoring Matrix (PSSM) (Jones 1999). The uniref90 database was used to generate the PSSM with three iterations and an E-value cut-off 0.01. The co-evolutionary information was computed using Gremlin (Kamisetty et al. 2013). The multiple sequence alignment (MSA) used by Gremlin was generated by HHblist (Remmert et al. 2012) with an E-value of 1 × 10^–10^ using four iterations. The variants were generated and clustered using PAM30 and the lowest-scored variant was chosen within each cluster by Rosetta. All scripts are provided in the supplementary information.

### Construction of variants

The vectors with substitutions were constructed by seamless cloning based on homologous recombination (Lu 2005). First, the open reading frames of V2 or V3 (Supplementary Information) were spliced into several fragments using the mutant site as the cut-off point. The mutant site was then introduced into primes (Table S4). The fragments were amplified by PCR with corresponding primers following with gel purification. For metabolite detection, the reconstructive vector pYES2-Trp was linearized with digestion of KpnI and SphI. The DNA purification products and linearized vector were recombined with In-Fusion HD Cloning kit (Takara) at 50 °C for 30 min. The product was transformed into *Escherichia coli* competent cells (DH5a) following by screening and identification of positive clones by sequence analysis. The yeast expression vector containing Myc tag (pYES2-Myc) was used to detect the expression of CYP87D20 variants by Western blotting. The variants were constructed using the same methods as the metabolite detection vector.

### Protein heterologous expression and metabolite extraction

The vectors pYES2-Trp containing different CYP87D20 variants were transformed into chassis strains (SC-Leu). These strains contained the OSC and CPR in the *S. cerevisiae* laboratory INVSc1 (Invitrogen) as previously described (Gietz and Schiestl 2007), such that it could produce the precursor (cucurbitadienol) (Shang et al. 2014). First, a single colony was inoculated into 5 mL of SC-Trp/Leu medium containing 2% glucose and grown overnight at 30 °C with shaking. The OD600 of the overnight culture was measured and a measured volume of cells was extracted to obtain an OD600 of 0.4 in 50 mL of induction medium (SC-Trp/Leu containing 2% galactose). The yeast cells were then cultured in the shaker at 30 °C for 200 rpm. After 48 h, the OD600 of the culture was measured and same volume of cells was extracted before being centrifuged at 1500 *g* at 4 °C. The pellet was re-suspended with alkali lysis buffer (20% KOH:50% ethanol = 1:1) for 1 h with intermittent shaking every 15 min. The lysate was extracted with 5 mL of petroleum ether three times. The upper superorganic phase was then gathered and dried with nitrogen gas.

### Metabolite detection by HPLC-APCI-qTOF

The metabolite extraction was dissolved in 5 mL methanol following ultrasonic treatment and purification by filtering through a 0.22 µm membrane. Chromatography was performed by an Agilent 1200 HPLC system. qTOF-MS was performed using an Agilent 6510 Q-TOF system equipped with atmospheric pressure chemical ionization (APCI) in positive ion model. An HHS T3 column with a 3.5 μm, 4.6 × 150 mm column was used (Waters, USA), with the temperature maintained at 35 °C. The mobile phase consisted of 0.1% formic acid aqueous solution (v/v, solvent A) and methanol/0.1% formic acid (v/v, solvent B). The flow rate was set at 1 mL/min, and the injection volume was 20 μL. The linear gradient with the following proportion of phase B (tmin, B%) was used: (1, 90), (3, 100), (10, 100), (12, 90), and (15, 90). The optimum MS conditions were documented as the following: corona current = 4 uA; capillary voltage = 5.0 kV; skimmer voltage = 65 V; segmented fragmentor = 135 V; gas temperature = 350 °C; vaporizer temperature = 400 °C; drying gas flow rate = 8 L/min; and nebulizer pressure = 60 psi. Nitrogen was used as the collision, drying, and nebulizer gas. MS spectra were acquired at 150–1000 *m/z* using an extended dynamic range and a scan rate of 1.5 spectra/s by varying collision energy with mass.

### Analysis of metabolite detection data

Three biological replicates with two technical replicates for each variant were performed to quantify metabolites. The standard substances, 11C-Cuol and 11C-20H-Cuol, were analyzed simultaneously for the standard curve (Zhou et al. 2016). 11H-Cuol is similar with 11C-Cuol, so they use the same standard curve. The data was analyzed by the software Profinder 10.00 which calculated the peak areas (Fernández-Ochoa et al. 2020). The extracted ion chromatography (EIC) of 11H-Cuol, 11C-Cuol, and 11C-20H-Cuol were 443.3884, 441.3709, and 457.3676 (*m/z*), respectively (Li et al. 2019). The yield of these products was calculated based on the standard curve, and the final yield was averaged with standard deviation.

### Extraction and detection of yeast total protein

The vectors pYES2-Myc containing different CYP87D20 variants were transformed into INVSc1. The positive single colony was inoculated with 5 mL of SC-Ura containing 2% glucose, and grown overnight at 30 °C with shaking. The OD600 of overnight culture was measured and a volume of cells was extracted to obtain an OD600 of 0.4, before being was transferred in 5 mL of induction medium (SC-Ura containing 2% galactose). The cells were grown at 30 °C with shaking for 12 h. The protein was extracted by acetone-trichloroacetic acid (TCA) (Niu et al. 2018). First, 5 OD yeast cells were collected by centrifuging at 1500 *g* for 5 min at 4 °C. Next, the pellet was re-suspended with 1 mL 0.25 M NaOH containing 1% β-mercaptoethanol and placed on ice for 10 min. 160 μL 50% TCA was added and mixed, and the solution remained on ice for another 10 min. The deposits were then collected by centrifuging at 14,000 *g* for 10 min at 4 °C, before removing the supernatant and re-suspending the precipitate in 1 mL of precool acetone. The mixture was then centrifuged at 14,000 *g* for 10 min at 4 °C, with the resulting supernatant removed. The precipitate was left to dry and boiled with 100 μL 1 × SDS protein loading buffer. 10 μL sample was used for Western blotting analysis with anti-Myc antibody. Yeast endogenous protein tubulin was used as the control for total protein quantification.

## Supplementary Information

Below is the link to the electronic supplementary material.

Supplementary file1 (DOCX 1954 KB)

Supplementary file2 (PDF 115 KB)

Supplementary file3 (PDF 337 KB)
