# Supplementary material for: Enhanced chemoselectivity of a plant cytochrome P450 through protein engineering of surface and catalytic residues
Source: aBIOTECH. 2021 Aug 10;2(3):215–25. doi: 10.1007/s42994-021-00056-z (PMC9590459; doi:10.1007/s42994-021-00056-z)
Supplement: Supplementary file 1 — Supplementary file1 (DOCX 1954 KB) [file 42994_2021_56_MOESM1_ESM.docx]

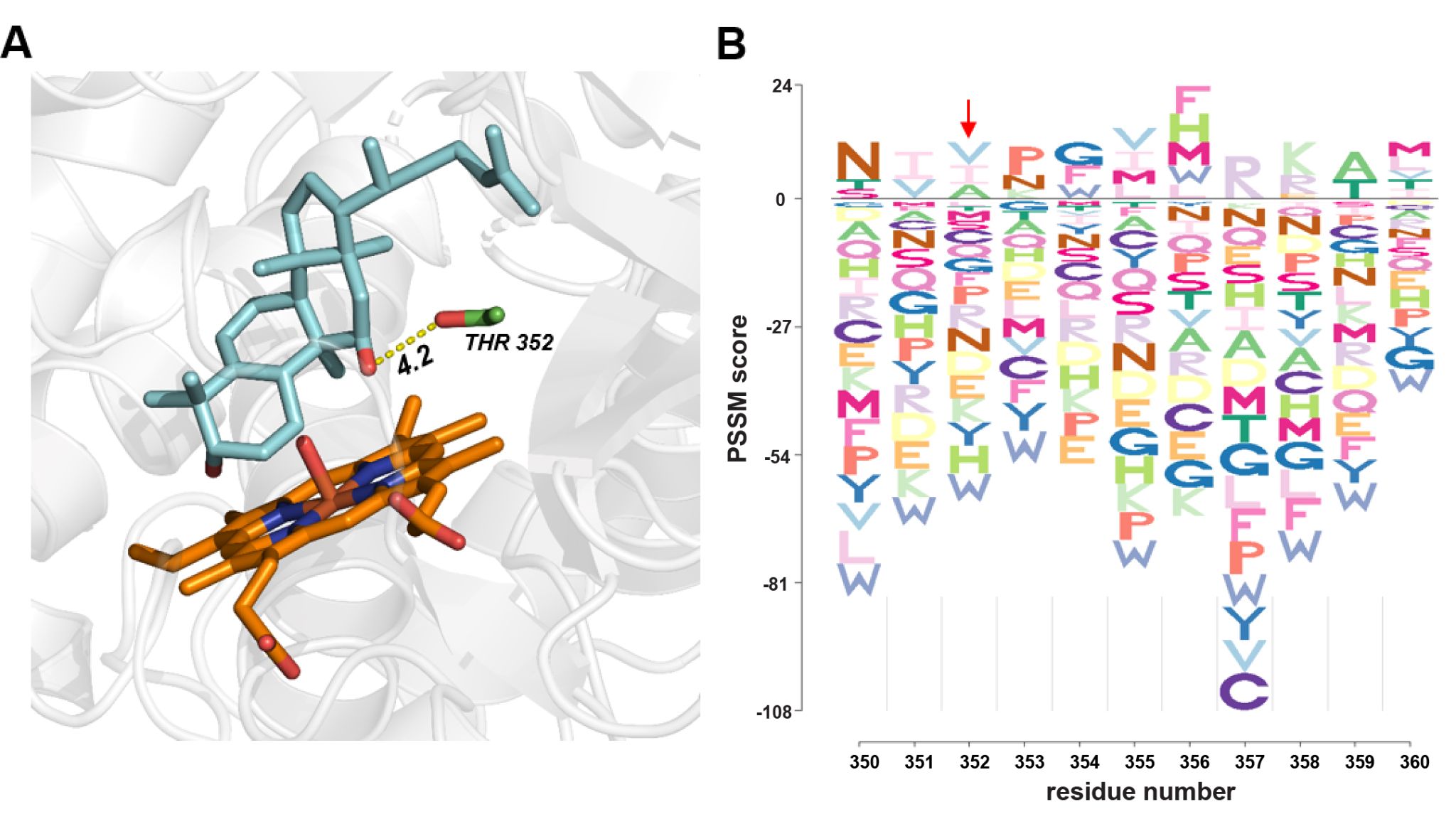


**Fig. S1** Computational model of the interaction between the C11 hydroxyl group of 11H-Cuol and T352, and PSSM score at position 352. **A**, The active site residue T352 and its interaction to the substrate. The P450 is shown in cartoon and the carbon atoms of the substrate 11H-Cuol are shown as cyan sticks; the heme group with a coordinating oxygen is shown in sticks with carbons copper colored, nitrogen blue, oxygen red, and iron orange. The figures were generated with PyMOL. **B**, Position Specific Scoring Matrices (PSSM) analysis of CYP87 family from position 350 to 360 where the log score is indicated as the size of the amino acid. The position 352 is indicated with a red arrow.


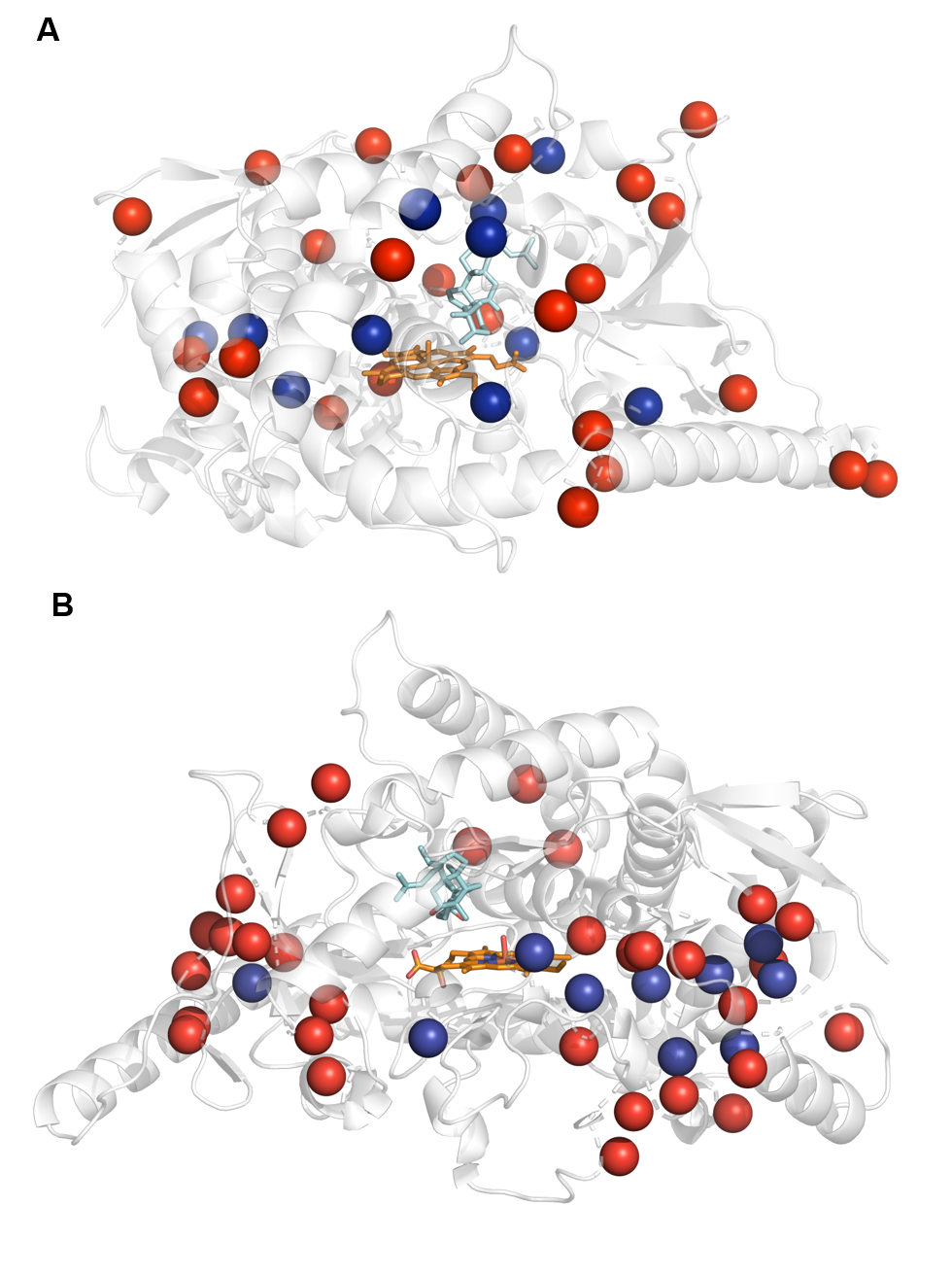


**Fig. S2** Illustration of the substitutions identified by phylogenetic analysis. **A**, Positions identified by PSSM. **B**, Positions identified by Gremlin. Red spheres, surface residue; Blue spheres, non-exposed residues. The P450 is shown in cartoon and the carbon atoms of the substrate 11H-Cuol are shown as cyan sticks; the heme group with a coordinating oxygen is shown in sticks with carbons copper colored, nitrogen blue, oxygen red, and iron orange. The figures were generated with PyMOL.


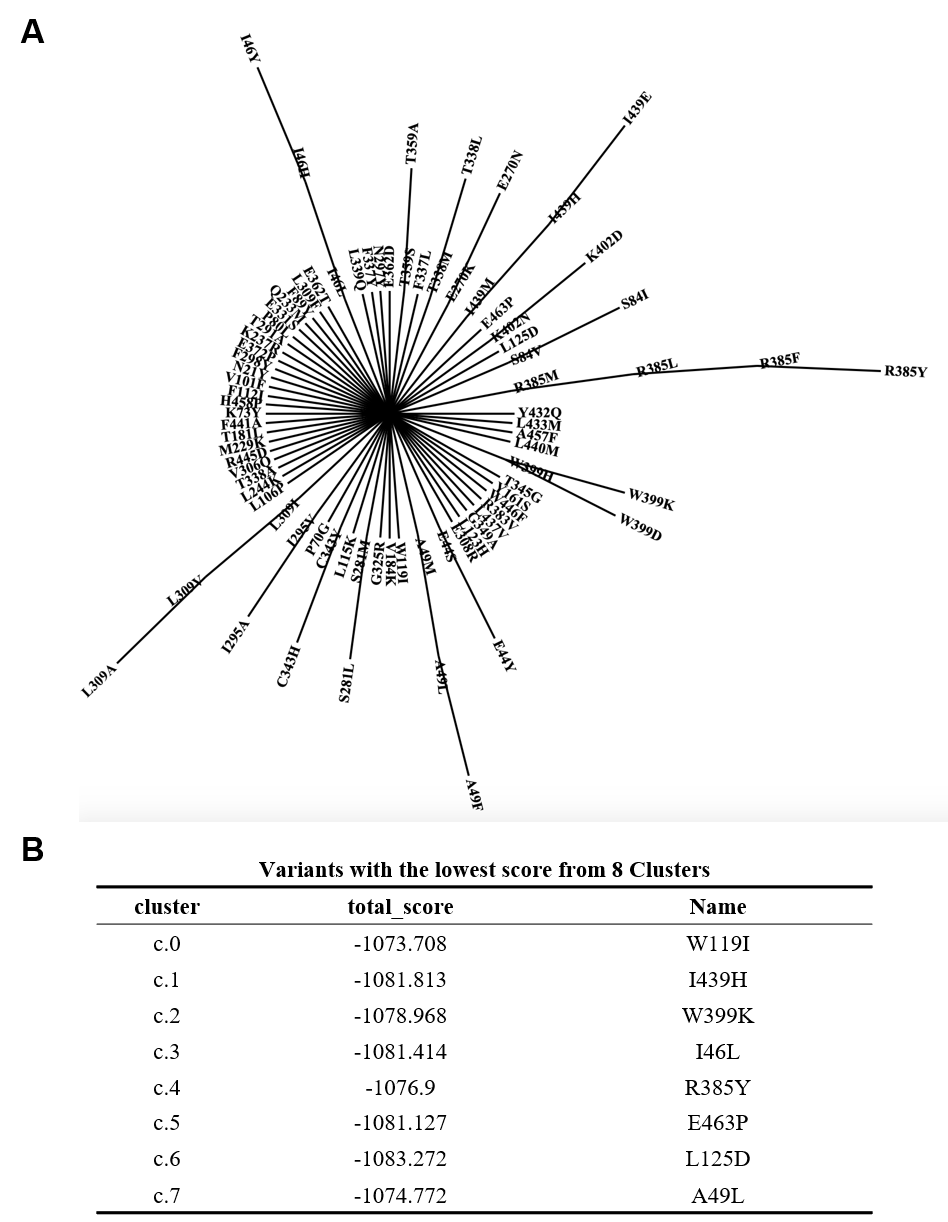


**Fig. S3** Clustering of variants and lowest scored cluster variants. **A**, The V2 variants were clustered using PhyML in Seaview for illustration. **B**, The variants were clustered using PAM30 using hierarchical clustering, and the lowest-scored variant was chosen within each cluster for experimental characterization.


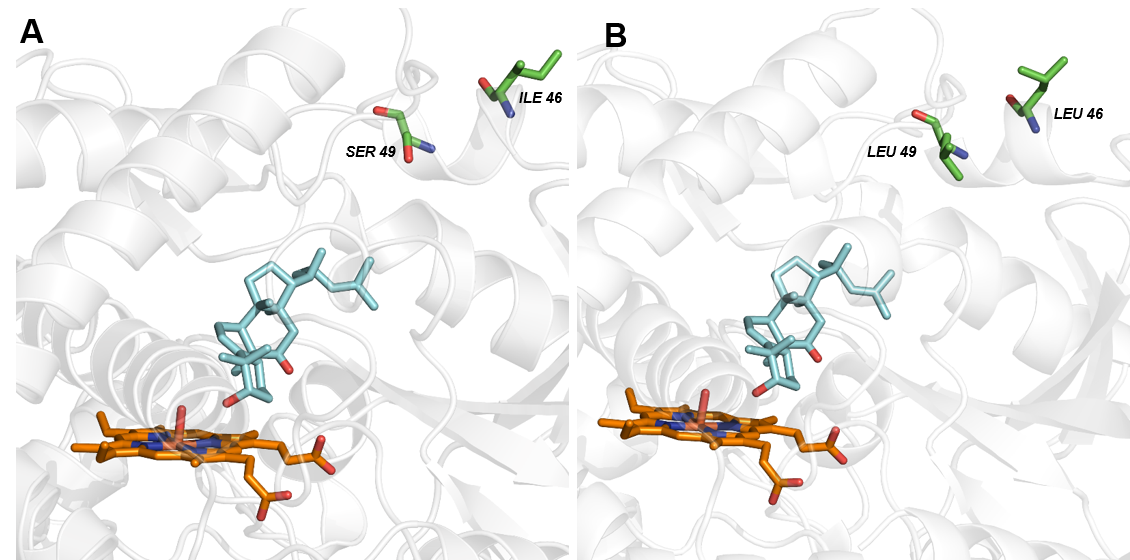


**Fig. S4** Computational model illustrating the surface position 46 and the semi-exposed position 49. before (**A**) and after mutation (**B**). The double mutation, I46L-S49L, may participate in the regulation of the substrate entry and orientation. The P450 is shown in cartoon and the carbon atoms of the substrate 11H-Cuol are shown as cyan sticks; the heme group with a coordinating oxygen is shown in sticks with carbons copper colored, nitrogen blue, oxygen red, and iron orange. The figures were generated with PyMOL.


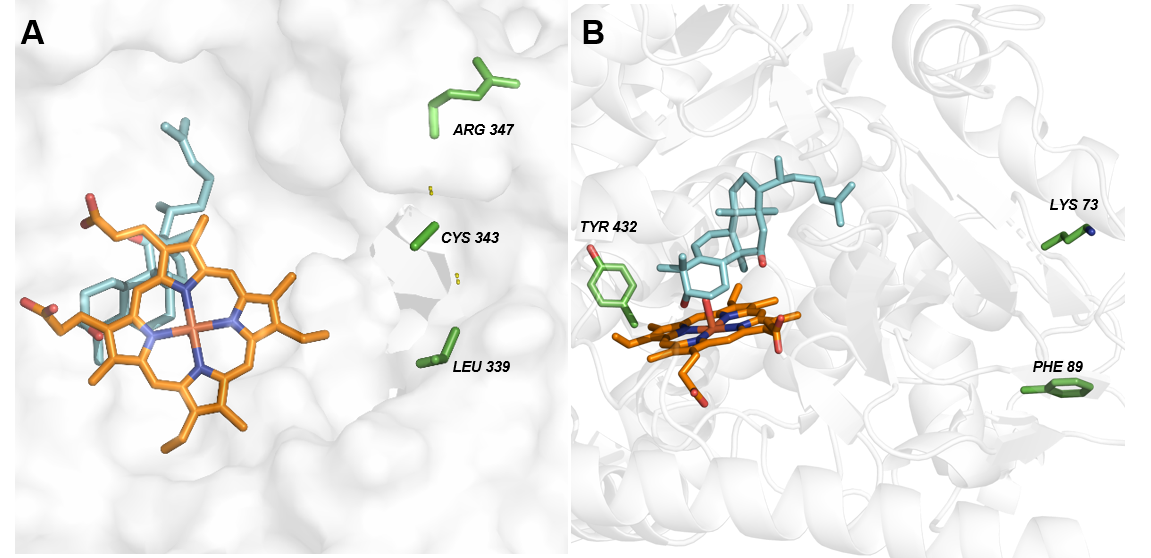
 **Fig. S5** Key amino acids identified during the protein engineering. **A**, The surface residue C343 with two surrounding amino acids, mutation of which to tyrosine improved the activity of the enzyme even though it is distal to the catalytic site. **B**, Positions of two surface residues (K73 and F89), and one active site residue (Y432), where each mutation abolished the activity of V3. The P450 is shown in cartoon and the carbon atoms of the substrate 11H-Cuol are shown as cyan sticks; the heme group with a coordinating oxygen is shown in sticks with carbons copper colored, nitrogen blue, oxygen red, and iron orange. The figures were generated with PyMOL.
