# Supplementary material for: Enhanced chemoselectivity of a plant cytochrome P450 through protein engineering of surface and catalytic residues
Source: aBIOTECH. 2021 Aug 10;2(3):215–25. doi: 10.1007/s42994-021-00056-z (PMC9590459; doi:10.1007/s42994-021-00056-z)
Supplement: Supplementary file 2 — Supplementary file2 (PDF 115 KB) [file 42994_2021_56_MOESM2_ESM.pdf]

Supplementary Information

S1. Score variants with Rosetta.

Each of the variants were scored with Rosetta

```
rosetta_scripts.static.linuxgccrelease -parser:protocol $xml @../flags -out:file:scorefile
../score/$prefix\.sc
```

with the following flags file

```
-run:preserve_header
-in:file:s model1.pdb
-extra_res_fa LIG.params
-extra_res_fa LG.params
-out:overwrite
-packing
  -use_input_sc
  -ex1
  -ex2
-no_optH false
-flip_HNQ
-no_his_his_pairE
-nstruct 1
-out:file:silent_struct_type binary
```

and the following xml-file

```
<ROSETTASCRIPTS>
   <SCOREFXNS>
      <ScoreFunction name="hard_rep" weights="ref2015">

      </ScoreFunction>
   </SCOREFXNS>
  <TASKOPERATIONS>

   <InitializeFromCommandline name="init"/>
   <LimitAromaChi2 name="limchi2"/>
   <RestrictToRepacking name="repack_only"/>
   <DesignAround name="da" design_shell="8.0" resnums="49A" repack_shell="6.0"
allow_design="0"/>

  </TASKOPERATIONS>

   <FILTERS>
   </FILTERS>
```

```
<MOVERS>

<MutateResidue name="mr49" target="49A" new_res="PHE"/>


# Minimization of complex - no design allowed
<TaskAwareMinMover name="min" bb="0" chi="1" jump="1" scorefxn="hard_rep"
task_operations="init"/>
# Packing of rotamers making sure no aromatic with chi2 of 90 degrees
<PackRotamersMover name="repack" task_operations="init,limchi2,repack_only,da"/>

<ParsedProtocol name="min_repack_min">
  <Add mover="min"/>
  <Add mover="repack"/>
  <Add mover="min"/>
</ParsedProtocol>

</MOVERS>

  <APPLY_TO_POSE>
  </APPLY_TO_POSE>

<PROTOCOLS>

  # Insert mutations
  <Add mover_name="mr49"/>

  <Add mover_name="min_repack_min"/>

</PROTOCOLS>

</ROSETTASCRIPTS>
```

S2. Generation of position specific scoring matrix.

The position specific scoring matrix was created using PSIBLAST 2.2.27+ with sequence from uniref90

```
path=/home/ubuntu/ncbi-blast-2.7.1+/bin
database=/home/ubuntu/uniref90
$path/psiblast -query $1 -db $database/uniref90_w_index.fasta -out_pssm my_protein.ckp -
evalue 0.01 -out_ascii_pssm ascii_mtx_file -out output_file -num_iterations 3
```

Which generates a scoring matrix.

S3. Multiple sequence alignment for Gremlin.

The sequence alignment necessary for Gremlin was generated using hhblists with uniclust30_2018_08 using a fasta-file as input with the following parameters

/hh-suite/build/bin/hhblits -o *OUTPUTNAME*.out -i *INPUTFILE*.fasta -oa3m *OUTPUTNAME*.a3m -d ./database/uniclust30_2018_08_hhsuite/uniclust30_2018_08/uniclust30_2018_08 -n 4 -e 1E-10 -mact 0.35 -maxfilt 100000000 -neffmax 20 -cpu 5 -nodiff -realign_max 10000000 -maxmem 64

which generates a multiple alignment in fasta-format used as input for Gremlin.

S4. The sequences of V2 and V3

V2: L48F-S49A-I61F-L120T-T352I-L356P

The nucleotide sequence for V2:

ATGTGGACGATCTTGCTCGGTTTGGCGACGTTGGCAATTGCCTACTATATTCATTGG
GTTAACAAATGGAAGGATTCTAAATTCAACGGAGTTTTGCCGCCGGGCACCATGGG
GCTGCCCCTCATCGGAGAAACCATTCAATTTGCTCGCCCTAGTGACTCCCTTGATGTT
CATCCTTTCTTTCAACGCAAAGTTAAAAGATATGGACCGATCTTCAAGACTTGTTTG
GCGGGAAGGCCGGTGGTGGTTTCAACGGATGCAGAGTTTAACCATTACATAATGCTC
CAAGAAGGAAGGGCCGTAGAAATGTGGTATTTGGATACACTCTCTAAATTCTTTGGC
CTTGACACTGAATGGACCAAAGCCCTTGGCCTCATCCACAAATACATTAGAAGCATT
ACTTTGAACCACTTTGGTGCTGAGTCCCTTCGTGAGCGTTTCCTTCCTCGTATCGAAG
AATCCGCTCGAGAAACCCTTCATTATTGGTCAACTCAAACCAGCGTTGAAGTCAAGG
AATCAGCCGCTGCGATGGTTTTCAGAACTTCGATTGTTAAGATGTTTAGTGAAGATT
CTAGTAAATTACTGACAGAAGGTCTCACTAAGAAGTTCACAGGACTTCTCGGAGGTT
TTCTCACCTTGCCTCTAAATTTGCCTGGCACTACCTATCATAAATGCATAAAGGACAT
GAAGCAAATCCAAAAGAAGCTAAAAGACATTTTAGAGGAAAGATTGGCTAAAGGG
GTTAAAATTGATGAAGATTTCTTGGGGCAAGCCATTAAAGATAAAGAATCTCAACA
ATTCATTTCAGAGGAATTCATTATCCAGTTGTTGTTTTCCATCAGCTTTGCTAGCTTT
GAGTCCATCTCTACCACTCTTACTTTGATTCTCAACTTCCTCGCCGATCACCCCGACG
TAGTGAAAGAATTGGAGGCTGAGCATGAGGCTATTAGAAAGGCAAGGGCAGATCCA
GATGGACCAATCACTTGGGAAGAATACAAATCCATGAATTTCACACTCAATGTCATC
TGTGAAACACTTAGGTTGGGAAGTGTAATACCTGCTTTGCCGAGGAAGACAACCAA
GGAAATTCAAATAAAAGGATACACAATTCCAGAAGGATGGACAGTAATGCTTGTGA
CCGCTTCTCGTCATAGAGATCCAGAAGTGTACAAGGATCCCGATACCTTCAATCCAT
GGCGTTGGAAGGAGTTGGACTCAATTACTATTCAAAAGAACTTCATGCCATTTGGGG
GAGGCTTAAGGCATTGTGCTGGTGCTGAATACTCTAAAGTCTATTTGTGCACTTTCCT
TCATATCCTTTTCACCAAATACAGATGGAGAAAACTAAAGGGAGGAAAGATTGCAA
GGGCTCATATATTGAGGTTTGAAGATGGGTTATATGTGAACTTCACTCCCAAGGAAT
GA

The amino acid sequence of V2:

MWTILLGLATLAIAYYIHWVNKWKDSKFNGVLPPGTMGLPLIGETIQFARPSDSLDVHP
FFQRKVKRYGPIFKTCLAGRPVVVSTDAEFNHYIMLQEGRAVEMWYLDTLSKFFGLDT
EWTKALGLIHKYIRSITLNHFGAESLRERFLPRIEESARETLHYWSTQTSVEVKESAAAM
VFRTSIVKMFSEDSSKLLTEGLTKKFTGLLGGFLTLPLNLPGTTYHKCIKDMKQIQKKLK
DILEERLAKGVKIDEDFLGQAIKDKESQQFISEEFIIQLLFSISFASFESISTTLTLILNFLADH
PDVVKELEAEHEAIRKARADPDGPITWEEYKSMNFTLNVICETLRLGSVIPALPRKTTKEI
QIKGYTIPEGWTVMLVTASRHRDPEVYKDPDTFNPWRWKELDSITIQKNFMPFGGGLRH
CAGAEYSKVYLCTFLHILFTKYRWRKLKGGKIARAHILRFEDGLYVNFTPKE

V3: L109F-F113L-E286A

The nucleotide sequence for V3:

ATGTGGACGATCTTGCTCGGTTTGGCGACGTTGGCAATTGCCTACTATATTCATTGG
GTTAACAAATGGAAGGATTCTAAATTCAACGGAGTTTTGCCGCCGGGCACCATGGG
GCTGCCCCTCATCGGAGAAACCATTCAACTTAGTCGCCCTAGTGACTCCCTTGATGT
TCATCCTTTCATTCAACGCAAAGTTAAAAGATATGGACCGATCTTCAAGACTTGTTT
GGCGGGAAGGCCGGTGGTGGTTTCAACGGATGCAGAGTTTAACCATTACATAATGC
TCCAAGAAGGAAGGGCCGTAGAAATGTGGTATTTGGATACATTCTCTAAATTCTTGG
GCCTTGACACTGAATGGCTCAAAGCCCTTGGCCTCATCCACAAATACATTAGAAGCA
TTACTTTGAACCACTTTGGTGCTGAGTCCCTTCGTGAGCGTTTCCTTCCTCGTATCGA
AGAATCCGCTCGAGAAACCCTTCATTATTGGTCAACTCAAACCAGCGTTGAAGTCAA
GGAATCAGCCGCTGCGATGGTTTTCAGAACTTCGATTGTTAAGATGTTTAGTGAAGA
TTCTAGTAAATTACTGACAGAAGGTCTCACTAAGAAGTTCACAGGACTTCTCGGAGG
TTTTCTCACCTTGCCTCTAAATTTGCCTGGCACTACCTATCATAAATGCATAAAGGAC
ATGAAGCAAATCCAAAAGAAGCTAAAAGACATTTTAGAGGAAAGATTGGCTAAAGG
GGTTAAAATTGATGAAGATTTCTTGGGGCAAGCCATTAAAGATAAAGAATCTCAAC
AATTCATTTCAGAGGAATTCATTATCCAGTTGTTGTTTTCCATCAGCTTTGCTAGCTT
TGCGTCCATCTCTACCACTCTTACTTTGATTCTCAACTTCCTCGCCGATCACCCCGAC
GTAGTGAAAGAATTGGAGGCTGAGCATGAGGCTATTAGAAAGGCAAGGGCAGATCC
AGATGGACCAATCACTTGGGAAGAATACAAATCCATGAATTTCACACTCAATGTCAT
CTGTGAAACACTTAGGTTGGGAAGTGTAACACCTGCTTTGTTGAGGAAGACAACCA
AGGAAATTCAAATAAAAGGATACACAATTCCAGAAGGATGGACAGTAATGCTTGTG
ACCGCTTCTCGTCATAGAGATCCAGAAGTGTACAAGGATCCCGATACCTTCAATCCA
TGGCGTTGGAAGGAGTTGGACTCAATTACTATTCAAAAGAACTTCATGCCATTTGGG
GGAGGCTTAAGGCATTGTGCTGGTGCTGAATACTCTAAAGTCTATTTGTGCACTTTC
CTTCATATCCTTTTCACCAAATACAGATGGAGAAAACTAAAGGGAGGAAAGATTGC
AAGGGCTCATATATTGAGGTTTGAAGATGGGTTATATGTGAACTTCACTCCCAAGGA
ATGA

The amino acid sequence of V3:

MWTILLGLATLAIAYYIHWVNKWKDSKFNGVLPPGTMGLPLIGETIQLSRPSDSLDVHPF
IQRKVKRYGPIFKTCLAGRPVVVSTDAEFNHYIMLQEGRAVEMWYLDTFSKFLGLDTE
WLKALGLIHKYIRSITLNHFGAESLRERFLPRIEESARETLHYWSTQTSVEVKESAAAMV
FRTSIVKMFSEDSSKLLTEGLTKKFTGLLGGFLTLPLNLPGTTYHKCIKDMKQIQKKLKDI
LEERLAKGVKIDEDFLGQAIKDKESQQFISEEFIIQLLFSISFASFASISTTLTLILNFLADHP

DVVKELEAEHEAIRKARADPDGPITWEEYKSMNFTLNVICETLRLGSVTPALLRKTTKEI
QIKGYTIPEGWTVMLVTASRHRDPEVYKDPDTFNPWRWKELDSITIQKNFMPFGGGLRH
CAGAEYSKVYLCTFLHILFTKYRWRKLKGGKIARAHILRFEDGLYVNFTPKE
