# Supplementary material for: Enhanced chemoselectivity of a plant cytochrome P450 through protein engineering of surface and catalytic residues
Source: aBIOTECH. 2021 Aug 10;2(3):215–25. doi: 10.1007/s42994-021-00056-z (PMC9590459; doi:10.1007/s42994-021-00056-z)
Supplement: Supplementary file 3 — Supplementary file3 (PDF 337 KB) [file 42994_2021_56_MOESM3_ESM.pdf]

Table S1. Potential postitions selected for the V2 engineering from PSSM

Position	Native	Substitution	PSSM Score Native	PSSM Score substitution	Position
21	N	Y	0	7	Surface
25	D	N	-1	6	Surface
39	L	W	3	11	Surface
75	C	N	-2	5	Surface
75	C	H	-2	8	Surface
77	A	F	0	7	Surface
95	L	Q	-1	8	Surface
106	L	P	0	8	Surface
115	L	K	-3	6	Surface
123	L	H	-2	6	Surface
125	L	D	-3	4	Surface
150	R	D	-2	6	Surface
161	Y	S	-4	5	Surface
184	V	K	-1	4	Surface
212	T	S	0	7	Surface
233	Q	M	-1	6	Surface
297	N	K	-2	7	Surface
297	N	Y	-2	5	Surface
339	L	Q	-1	6	Surface
343	C	N	-2	7	Surface
362	E	D	1	8	Surface
385	R	Y	-4	4	Surface
394	D	L	-3	4	Surface
439	I	H	0	8	Surface
457	A	F	-2	6	Surface
463	E	P	-2	7	Surface
467	Y	H	0	8	Surface
49	A	L	-4	4	Few exposed，Near the tail of substrate
101	V	F	2	9	Around Heme
112	F	I	-3	6	Near the tail of substrate
112	F	L	-3	4	Near the tail of substrate
119	W	I	-3	5	Around Heme
181	T	L	-3	4	Interior
229	M	R	-4	8	Interior
229	M	K	-4	3	Interior
281	S	L	-2	5	Interior
281	S	M	-2	5	Interior
383	R	V	-3	6	Interior
432	Y	E	-3	5	Interior
441	F	V	-3	7	Interior
458	H	P	-3	9	Interior

Table S2. Potential postitions selected for the V2 engineering from Gremlin

Position	Native	Substitution	Position
44	E	N,S,Y	Surface
46	I	H,L.Y	Surface
70	P	G,K	Surface
73	K	F,T,Y	Surface
80	P	L	Surface
84	S	I,L,V,A	Interior
89	F	D,I,L,Y	Surface
244	L	K	Surface
270	E	R,K,N,L	Surface
298	F	Y	Surface
299	L	I	Few exposed
306	V	Q,E	Few exposed
308	E	R,Q	Surface
325	G	R	Few exposed
331	E	A,S	Surface
337	F	L,Y	Surface
343	C	N,H,Y	Surface
358	K	E,M,V	Surface
362	E	T	Surface
369	T	R	Surface
372	E	P	Surface
373	G	D	Surface
374	W	T	Surface
399	W	H,K,D	Surface
402	K	N,D,E	Surface
439	I	E,M,Y	Surface
445	R	D,E	Surface
446	W	F	Surface
237	K	A,R	Interior
291	T	A	Around heme
295	I	A,C,G,S,M,V	Around heme
309	L	V,L,F,I	Interior
338	T	A,L,M	Interior
345	T	A,G	Interior
349	G	A	Interior
359	T	S	Interior
383	R	T	Interior
433	L	M,V	Interior
437	L	T,V	Interior
440	L	M	Interior
441	F	A,V	Interior

Table S3. Variants for V3

Position	Native	Substitution	Source	Position
73	K	Y	Gremlin	Surface
89	F	D	Gremlin	Surface
125	L	D	PSSM	Surface
343	C	Y	PSSM	Surface
383	R	T	PSSM	Interior
399	W	D	Gremlin	Surface
432	Y	E	PSSM	Interior

Table S4. The primers of PCR and sequencing

Variants	Mutation site	Primers		Templates	Vector Construction	
V2	L48F-S49A-I61F-L120T-K352I-L356P	WT-F	GACTCACTATAGGGAATATTAAGCTTGGTACCATGTGGACGATCTTGCTC	L48F-S49A-I61F-L120T-T352K-L356P	Fragment1	Seamless Clone
		V2-1R	GGCAAAGCAGGTATTACACTTCCCAACCTAAGTGTTTCACAGATG			
		V2-2F	GGGAAGTGTAATACCTGCTTTGCCGAGGAAGACAACCAAGG		Fragment2	Seamless Clone
		WT-R	GATGCGGCCCTCTAGATGCATGCTCATTCCTTGGGAGTGAAGTTC			
V2-I46L-A49L	L48F-S49A-I61F-L120T-K352I-L356P_I46L_A49L	WT-F	GACTCACTATAGGGAATATTAAGCTTGGTACCATGTGGACGATCTTGCTC	L48F-S49A-I61F-L120T-K352I-L356P	Fragment1	Seamless Clone
		V3-1R	GGGCGAAGAAATTGAAGGGTTTCTCCGATGAGGGGCAGCCCCATGG			
		V3-2F	GAGAAACCCTTCAATTTCTTCGCCCTAGTGACTCCCTTGATGTTCATCC		Fragment2	Seamless Clone
		WT-R	GATGCGGCCCTCTAGATGCATGCTCATTCCTTGGGAGTGAAGTTC			
V2-W119I-L125D	L48F-S49A-I61F-L120T-K352I-L356P_W119I_L125D	WT-F	GACTCACTATAGGGAATATTAAGCTTGGTACCATGTGGACGATCTTGCTC	L48F-S49A-I61F-L120T-K352I-L356P	Fragment1	Seamless Clone
		V4-1R	GTACACTTCTGGATCATAATGACGAGAAGCGGTCACAAGCATTAC			
		V4-2F	CTCGTCATTATGATCCAGAAGTGTACAAGGATCCCGATACCTTC		Fragment2	Seamless Clone
		WT-R	GATGCGGCCCTCTAGATGCATGCTCATTCCTTGGGAGTGAAGTTC			
V2-R385Y	L48F-S49A-I61F-L120T-K352I-L356P_R385Y	WT-F	GACTCACTATAGGGAATATTAAGCTTGGTACCATGTGGACGATCTTGCTC	L48F-S49A-I61F-L120T-K352I-L356P	Fragment1	Seamless Clone
		V5-1R	GGATGTCGCCAAGGGCTTTGGTGATTTCAGTGTCAAGGCCAAAGAATTTAG			
		V5-2F	CTGAAATCACCAAAGCCCTTGGCGACATCCACAAATACATTAGAAGCATTAC		Fragment2	Seamless Clone
		WT-R	GATGCGGCCCTCTAGATGCATGCTCATTCCTTGGGAGTGAAGTTC			
V2-W399K	L48F-S49A-I61F-L120T-K352I-L356P_W399K	WT-F	GACTCACTATAGGGAATATTAAGCTTGGTACCATGTGGACGATCTTGCTC	L48F-S49A-I61F-L120T-K352I-L356P	Fragment1	Seamless Clone
		V6-1R	CTCCTTCCAACGCTTTGGATTGAAGGTATCGGGATCCTTG			
		V6-2F	CAATCCAAAGCGTTGGAAGGAGTTGGACTCAATTACTATTC		Fragment2	Seamless Clone
		WT-R	GATGCGGCCCTCTAGATGCATGCTCATTCCTTGGGAGTGAAGTTC			
V2-I439H	L48F-S49A-I61F-L120T-K352I-L356P_I439H	WT-F	GACTCACTATAGGGAATATTAAGCTTGGTACCATGTGGACGATCTTGCTC	L48F-S49A-I61F-L120T-K352I-L356P	Fragment1	Seamless Clone
		V7-1R	GCACTTTCCTTCATCACCTTTTCACCAAATACAGATGGAGAAAAC			
		V7-2F	GGTGAAAAGGTGATGAAGGAAAGTGCACAAATAGACTTTAGAG		Fragment2	Seamless Clone
		WT-R	GATGCGGCCCTCTAGATGCATGCTCATTCCTTGGGAGTGAAGTTC			
V2-E463P	L48F-S49A-I61F-L120T-K352I-L356P_E463P	WT-F	GACTCACTATAGGGAATATTAAGCTTGGTACCATGTGGACGATCTTGCTC	L48F-S49A-I61F-L120T-K352I-L356P	Full-length Fragment	Seamless Clone
		V8-R	GGCCCTCTAGATGCATGCTCATTCCTTGGGAGTGAAGTTCACATATAACCCATCTGGAAAC			
V3	L109F-F113L-E286A	WT-F	GACTCACTATAGGGAATATTAAGCTTGGTACCATGTGGACGATCTTGCTC	WT	Fragment1	Seamless Clone
		V9-1R	GGCCCAAGAATTTAGAGAATGTATCCAAATACCACATTTCTACG			
		V9-2F	GGATACATTCTCTAAATTCTTGGGCCTTGACACTGAATGGCTC		Fragment2	Seamless Clone
		V9-2R	GTAGAGATGGACGCAAAGCTAGCAAAGCTGATGGAAAACAAC			
		V9-3F	GCTAGCTTTGCGTCCATCTCTACCACTCTTACTTTGATTCTC		Fragment3	Seamless Clone
		WT-R	GATGCGGCCCTCTAGATGCATGCTCATTCCTTGGGAGTGAAGTTC			
V3-C343Y	L109F-F113L-E286A_C343Y	WT-F	GACTCACTATAGGGAATATTAAGCTTGGTACCATGTGGACGATCTTGCTC	L109F-F113L-E286A	Fragment1	Seamless Clone
		V10-1R	CCTAAGTGTTTCATAGATGACATTGAGTGTGAAATTCATGGATTTGTATTC			
		V10-2F	CACTCAATGTCATCTATGAAACACTTAGGTTGGGAAGTGTAACACCTGCTTTG		Fragment2	Seamless Clone
		WT-R	GATGCGGCCCTCTAGATGCATGCTCATTCCTTGGGAGTGAAGTTC			
V3-K73Y	L109F-F113L-E286A_K73Y	WT-F	GACTCACTATAGGGAATATTAAGCTTGGTACCATGTGGACGATCTTGCTC	L109F-F113L-E286A	Fragment1	Seamless Clone
		V11-1R	CGCCAAACAAGTGTAGAAGATCGGTCCATATCTTTTAACTTTGCG			
		V11-2F	GACCGATCTTCTACACTTGTTTGGCGGGAAGGCCGGTGGTGGTTTC		Fragment2	Seamless Clone
		WT-R	GATGCGGCCCTCTAGATGCATGCTCATTCCTTGGGAGTGAAGTTC			
V3-F89D	L109F-F113L-E286A_F89D	WT-F	GACTCACTATAGGGAATATTAAGCTTGGTACCATGTGGACGATCTTGCTC	L109F-F113L-E286A	Fragment1	Seamless Clone
		V12-1R	GCATTATGTAATGGTTATCCTCTGCATCCGTTGAAACCACCACCGGCC			
		V12-2F	CGGATGCAGAGGATAACCATTACATAATGCTCCAAGAAGGAAGGGCCG		Fragment2	Seamless Clone
		WT-R	GATGCGGCCCTCTAGATGCATGCTCATTCCTTGGGAGTGAAGTTC			
V3-Y432E	L109F-F113L-E286A_Y432E	WT-F	GACTCACTATAGGGAATATTAAGCTTGGTACCATGTGGACGATCTTGCTC	L109F-F113L-E286A	Fragment1	Seamless Clone
		V13-1R	GGAAAGTGCACAACTCGACTTTAGAGTATTCAGCACCAGCACAATG			
		V13-2F	CTAAAGTCGAGTTGTGCACTTTCCTTCATATCCTTTTCACCAAATAC		Fragment2	Seamless Clone
		WT-R	GATGCGGCCCTCTAGATGCATGCTCATTCCTTGGGAGTGAAGTTC			
V3-L125D	L109F-F113L-E286A_L125D	WT-F	GACTCACTATAGGGAATATTAAGCTTGGTACCATGTGGACGATCTTGCTC	L109F-F113L-E286A	Fragment1	Seamless Clone
		V14-1R	GTATTTGTGGATGTCGCCAAGGGCTTTGAGCCATTCAGTGTCAAGGCCC			
		V14-2F	GCCCTTGGCGACATCCACAAATACATTAGAAGCATTACTTTGAACCAC		Fragment2	Seamless Clone
		WT-R	GATGCGGCCCTCTAGATGCATGCTCATTCCTTGGGAGTGAAGTTC			
V3-R383T	L109F-F113L-E286A_R383T	WT-F	GACTCACTATAGGGAATATTAAGCTTGGTACCATGTGGACGATCTTGCTC	L109F-F113L-E286A	Fragment1	Seamless Clone
		V15-1R	CTGGATCTCTATGAGTAGAAGCGGTCACAAGCATTACTGTCCATCC			
		V15-2F	GACCGCTTCTACTCATAGAGATCCAGAAGTGTACAAGGATCCCGATAC		Fragment2	Seamless Clone
		WT-R	GATGCGGCCCTCTAGATGCATGCTCATTCCTTGGGAGTGAAGTTC			
V3-W399D	L109F-F113L-E286A_W399D	WT-F	GACTCACTATAGGGAATATTAAGCTTGGTACCATGTGGACGATCTTGCTC	L109F-F113L-E286A	Fragment1	Seamless Clone
		V16-1R	CCTTCCAACGGTCTGGATTGAAGGTATCGGGATCCTTGTACAC			
		V16-2F	CCTTCAATCCAGACCGTTGGAAGGAGTTGGACTCAATTACTATTC		Fragment2	Seamless Clone
		WT-R	GATGCGGCCCTCTAGATGCATGCTCATTCCTTGGGAGTGAAGTTC			
V2-I46L-A49L-C343Y	L48F-S49A-I61F-L120T-K352I-L356P_I46L_A49L_C343Y	WT-F	GACTCACTATAGGGAATATTAAGCTTGGTACCATGTGGACGATCTTGCTC	3F-S49A-I61F-L120T-T352K-L356P-I46L-A4	Fragment1	Seamless Clone
		V17-1R	CCCAACCTAAGTGTTTCATAGATGACATTGAGTGTGAAATTCATGGATTTG			
		V17-2F	CAATGTCATCTATGAAACACTTAGGTTGGGAAGTGTAATACCTGCTTTG		Fragment2	Seamless Clone
		WT-R	GATGCGGCCCTCTAGATGCATGCTCATTCCTTGGGAGTGAAGTTC			
V3-C343Y-I46L	L109F-F113L-E286A_C343Y_I46L	WT-F	GACTCACTATAGGGAATATTAAGCTTGGTACCATGTGGACGATCTTGCTC	L109F-F113L-E286A-C343Y	Fragment1	Seamless Clone
		V18-1R	GAGAAAGTTGAAGGGTTTCTCCGATGAGGGGCAGCCCCATGGTG			
		V18-2F	CGGAGAAACCCTTCAACTTTCTCGCCCTAGTGACTCCCTTGATG		Fragment2	Seamless Clone
		WT-R	GATGCGGCCCTCTAGATGCATGCTCATTCCTTGGGAGTGAAGTTC			
V3-C343Y-S49L	L109F-F113L-E286A_C343Y_S49L	WT-F	GACTCACTATAGGGAATATTAAGCTTGGTACCATGTGGACGATCTTGCTC	L109F-F113L-E286A-C343Y	Fragment1	Seamless Clone
		V19-1R	CACTAGGGCGAAGAAGTTGAATGGTTTCTCCGATGAGGGGCAGC			
		V19-2F	CCATTCAACTTCTTCGCCCTAGTGACTCCCTTGATGTTCATCCTTTC		Fragment2	Seamless Clone
		WT-R	GATGCGGCCCTCTAGATGCATGCTCATTCCTTGGGAGTGAAGTTC			
